# Tumor Lymphatic Interactions Induce CXCR2-CXCL5 Axis and Alter Cellular Metabolism and Lymphangiogenic Pathways to Promote Cholangiocarcinoma

**DOI:** 10.3390/cells10113093

**Published:** 2021-11-09

**Authors:** Sukanya Roy, Subhashree Kumaravel, Priyanka Banerjee, Tori K. White, April O’Brien, Catherine Seelig, Rahul Chauhan, Burcin Ekser, Kayla J. Bayless, Gianfranco Alpini, Shannon S. Glaser, Sanjukta Chakraborty

**Affiliations:** 1 Department of Medical Physiology, College of Medicine, Texas A&M University Health Science Center, Bryan, TX 77807, USA; su_15@tamu.edu (S.R.); subhashree28.s@gmail.com (S.K.); pbanerjee@exchange.tamu.edu (P.B.); tkshepperd@tamu.edu (T.K.W.); aprilobrien@tamu.edu (A.O.); cat.2199@tamu.edu (C.S.); rahul.chauhan@tamu.edu (R.C.); sglaser@tamu.edu (S.S.G.); 2 Department of Surgery, Division of Transplant Surgery, Indiana University School of Medicine, Indianapolis, IN 46202-3082, USA; bekser@iupui.edu; 3 Department of Molecular and Cellular Medicine, College of Medicine, Texas A&M University Health Science Center, Bryan, TX 77807, USA; kaylajb@tamu.edu; 4 Department of Medicine, Division of Gastroenterology and Hepatology, Indiana University, Indianapolis, IN 46202-3082, USA; galpini@iu.edu; 5 Richard L. Roudebush VA Medical Center, Indianapolis, IN 46202-3082, USA

**Keywords:** chemokines, liver cancer, lymphatic metastasis, inflammation, bioenergetics

## Abstract

Cholangiocarcinoma (CCA), or cancer of bile duct epithelial cells, is a very aggressive malignancy characterized by early lymphangiogenesis in the tumor microenvironment (TME) and lymph node (LN) metastasis which correlate with adverse patient outcome. However, the specific roles of lymphatic endothelial cells (LECs) that promote LN metastasis remains unexplored. Here we aimed to identify the dynamic molecular crosstalk between LECs and CCA cells that activate tumor-promoting pathways and enhances lymphangiogenic mechanisms. Our studies show that inflamed LECs produced high levels of chemokine CXCL5 that signals through its receptor CXCR2 on CCA cells. The CXCR2-CXCL5 signaling axis in turn activates EMT (epithelial-mesenchymal transition) inducing MMP (matrix metalloproteinase) genes such as GLI, PTCHD, and MMP2 in CCA cells that promote CCA migration and invasion. Further, rate of mitochondrial respiration and glycolysis of CCA cells was significantly upregulated by inflamed LECs and CXCL5 activation, indicating metabolic reprogramming. CXCL5 also induced lactate production, glucose uptake, and mitoROS. CXCL5 also induced LEC tube formation and increased metabolic gene expression in LECs. In vivo studies using CCA orthotopic models confirmed several of these mechanisms. Our data points to a key finding that LECs upregulate critical tumor-promoting pathways in CCA via CXCR2-CXCL5 axis, which further augments CCA metastasis.

## 1. Introduction

CCA is the second most common type of liver cancer that involves the extra- and intrahepatic bile ducts. CCA is very aggressive with a dismal prognosis and a post-metastasis diagnosis five-year survival rate of only 2% [1]. Early diagnosis of CCA is challenging and treatment options are limited [2,3]. Tumor-associated growth of new lymphatic vessels (i.e., lymphangiogenesis) predicts unfavorable prognosis of CCA with tumor metastasis to the draining LNs as the primary prognostic indicator of tumor aggressiveness [4]. A high degree of both peri- and intra-tumoral lymphangiogenesis in the early stages of CCA indicates greater LN metastasis that makes surgical resection a significant challenge [5]. Recent evidence indicates that a complex interaction between tumor cells and LECs takes place, but their mechanisms remain grossly understudied [6]. Traditional chemotherapeutic regimens often do not target the lymphatics, thus making complete therapeutic inhibition of metastatic tumors a critical challenge. LECs have significant immunomodulatory roles, and tumor cells co-opt several mechanisms normally used by immune cells to traffic through lymphatics and LNs [7,8,9]. Thus, understanding the interplay between LECs and CCA could have a significant impact on therapeutic modalities.

The inflamed CCA TME is significantly pro-lymphangiogenic [10] and several lymphangiogenic factors such as high Vascular Endothelial Growth Factor C (VEGF-C) expression observed in iCCA (intrahepatic CCA) has been correlated to poor survival rates and enhanced lymphangiogenesis [11]. A high level of platelet derived growth factor D (PDGF-D) secreted from CCA cells promoted VEGF-C secretion from cancer-associated fibroblasts and enhanced lymphangiogenesis and LN metastasis [12]. Thus, various pro-lymphangiogenic factors in CCA TME sets the stage for the high CCA lymphangiogenesis and correlates with increased metastasis [3]. Activated LECs in an inflammatory TME, secrete cytokines and chemokines, which induces cancer cell growth and metastasis [13,14] but these pathways have not been studied in CCA. We hypothesized that an active crosstalk exists between CCA and LECs that is further instigated by the inflamed CCA TME, which results in high CCA LN metastasis and increased tumor-related lymphangiogenesis. One such axis is mediated by C-X-C motif chemokine receptor 2 (CXCR2), a typical G-protein-coupled receptor that is shown to be associated with chemoresistance in several cancers [15,16]. CXCR2 binds chemokines containing the glutamic acid-leucine-arginine (ELR) motif such as CXCL5 and CXCL8 that are involved in angiogenesis and also promote tumor progression [17]. CXCL5 signaling through the cognate receptor CXCR2 induces pro-inflammatory changes in the TME of different cancer types and augments cancer metastases through upregulating EMT pathways such as extracellular signaling-regulated kinase 1/2 (ERK1/2), glycogen synthase kinase 3 beta (GSK3 β), and SNAIL family transcriptional repressor 1 (Snail) [15,18]. Further, metabolic reprogramming of tumor cells and enhancement of glycolysis acts as a crucial step in EMT activation in cancer [19]. We have previously demonstrated that LECs significantly modulate cellular bioenergetics in head and neck cancers [14]. However, the role of the LECs in influencing tumor metabolism in CCA has not been previously elucidated.

In these studies, the following primary aims were addressed: (1) to delineate the molecular crosstalk mediated by specific chemokine/chemokine receptor between CCA and LECs and identification of a signaling axis that directly enhances CCA migration as well as promotes pro-lymphangiogenic pathways; and (2) evaluate the role of inflamed LECs on metabolic reprograming and alteration of metabolic pathways that promote tumor growth. In this study, we have made the interesting observation that CCA-LEC interactions significantly altered CCA cellular bioenergetics and also promoted lymphangiogenic mechanisms. Thus, we explore the role of CXCR2-CXCL5 signaling in mediating the crosstalk between CCA cells and LECs in the context of CCA growth, progression, metabolism and CCA related lymphangiogenesis.

## 2. Materials and Methods

### 2.1. Ethics Statement

All experiments were carried out in accordance with policies and approval of Texas A&M University Institutional Biosafety Committee and Institutional Animal Care and Use Committee.

### 2.2. Animal Models

All mice were housed in a temperature-controlled environment, with 12:12 h light-dark cycles and access ad libitum to water and standard mouse chow. CCA syngeneic orthotopic model: C57BL/6 wild-type (WT) mice were purchased from Charles River Laboratories (Wilmington, MA, USA). For generation of the orthotopic CCA tumor model, male C57/BL6 mice were injected with SB1 cells (1.1 × 10^6^) (a generous gift from Dr. Gregory Gores, Mayo Clinic, Rochester, MN, USA) into the upper lobe of each liver as described previously [20]. The mouse CCA SB1 cells are phenotypically similar to human CCA [20]. Cells were grown in plain DMEM supplemented with 10% FBS, and in the control animals, plain DMEM media was injected into the upper lobe of liver. The animals were observed for 3–4 weeks, before being euthanized. Tissues (liver tumors, normal liver and liver LNs) were isolated from the tumor bearing and control animals, respectively, and processed for immunofluorescence, RNA and protein isolation.

### 2.3. Cell Culture and Treatments

Primary human LECs (HLECs) from different de-identified donors were purchased from Promocell (Heidelberg, Germany) and grown in complete endothelial growth medium (EGM-MV2; Promocell, Heidelberg, Germany) [14]. The extrahepatic CCA cell line Mz-ChA-1 was a gift from Dr. J. Gregory Fitz (UT Southwestern, Dallas, TX, USA) and cultured as previously described [21]. The intrahepatic CCA cell lines HuCC-T1 and CCLP1 were gifts from Dr. Anthony J. Demetris (University of Pittsburgh, Pittsburgh, PA, USA) and cultured as described [22,23]. Short tandem repeats (STR) analysis from ATCC was carried out for cell line authentication. Cell lines were also routinely evaluated for mycoplasma contamination. For the collection of conditioned medium (CM), CCA cells (HuCC-T1, Mz-ChA-1, and CCLP1) were treated with plain RPMI for 24 h and subsequently the media was collected and centrifuged (1000× *g*, 5 min) to remove cell debris. LEC-CM was collected by growing LECs in plain EGM-MV2 (without any growth factors) for 48 h and centrifuged as described earlier. To induce LEC inflammation, LECs were treated with lipopolysaccharide (LPS); 100 ng/mL) purchased from Sigma Aldrich (St. Louis, MO, USA). For LPS-LEC-CM, LECs were stimulated with LPS (100 ng/mL) for 24 h. After 24 h, the LPS containing culture medium was removed and replaced with EGM-MV2 (supplemented with 5% FBS) (5% EGM) and left for another 24 h. The conditioned medium was then collected and centrifuged as described earlier [14]. All CM was either used immediately or stored at −80 °C for later use. Recombinant CXCL5 (rhCXCL5) and CXCR2 inhibitor (SB225002) were purchased from R&D Systems (Minneapolis, MN, USA) while ERK inhibitor PD98059 (PD) was purchased from Biomol Research Laboratories Inc. (PA, USA). CCA and LECs were stimulated with CXCL5 (20–50 nM) with or without pre-treatment with SB225002 (SB) (1 μM) and PD98059 (250 nM) for 2 h. All other chemicals and reagents were from Sigma Aldrich unless otherwise stated.

### 2.4. RNA Isolation and Real Time Quantitative Reverse Transcriptase PCR (qPCR)

Total RNA was isolated from human CCA cell lines and human LECs after treatments as described above using PureLink RNA Mini kit (Thermo Fisher Scientific, Waltham, MA, USA) (according to the manufacturer’s instructions). The quality and quantity of isolated RNA were verified by NanoDrop (NanoDrop Technologies, Wilmington, DE, USA) and subsequently cDNA was prepared using Maxima H Minus cDNA Synthesis kit from Life Technologies (Carlsbad, CA, USA). qPCR was performed using SYBR Green kit (Applied Biosystems, Waltham, MA, USA) for cytokines, chemokines, inflammatory markers, EMT-inducing markers and MMPs in a real-time thermal cycler (ABI Prism 7900HT sequence detection system; Applied Biosystems, CA, USA). All experiments were performed at least in triplicate. Primers for the above-mentioned genes were obtained from Sigma-Aldrich. qPCR data analysis was performed using the 2^−ΔΔCt^ method using RPL-19 as the housekeeping gene. Primer sequences have been provided in Table 1 below.

### 2.5. Cell Lysis and Western Blot

CCA cells were treated with LEC-CM or LPS-LEC-CM or with CXCL5, CXCL5 + SB225002 as above. Cells were lysed with M-PER (Thermo Fisher Scientific, Waltham, MA, USA) lysis buffer and protein quantification performed using Pierce BCA Protein Assay kit (Thermo Fisher Scientific, Waltham, MA, USA). Bolt 4–12% Bis-Tris gel (Invitrogen, Thermo Fisher Scientific, Waltham, MA, USA) was used for protein separation and subsequently were transferred onto a nitrocellulose membrane (Invitrogen, Thermo Fisher Scientific, Waltham, MA, USA) and probed for CXCR2(#ab217314, Abcam Inc, Cambridge, MA, USA), pERK (#9101,) and total ERK (#9102) were from Cell Signaling Technology (Danvers, MA, USA). All antibodies were used at 1:1000 dilution. Following corresponding secondary antibody incubation, proteins were detected using Chemiluminescence Detection kit (Pierce, Thermo Fisher Scientific, Waltham, MA, USA) and imaged with ImageQuant LAS 4000 system (GE Healthcare, Chicago, IL, USA). Densitometric analysis of bands was performed using ImageJ software version 1.52a (NIH, Bethesda, MD, USA), as described previously [24].

### 2.6. Boyden Chamber Assay

Migration of HuCC-T1 and Mz-ChA-1 cells were assessed by Boyden Chamber Assay. CCA cells (1 × 10^5^) were plated in 8.0 μM pore PTE standing inserts (Millipore, Burlington, MA, USA) placed in a 24 well plate and migration of CCA cells in response to LEC-CM and LEC-LPS-CM was measured. For determining the chemotactic role of CXCL5 and the effect of suppression of the CXCR2-CXCL5 axis in CCA migration, CCA cells were pre-treated with or without SB225002 (1 μM) for two hours and allowed to migrate towards plain DMEM/RPMI containing CXCL5 recombinant protein (20–50 nM). Cells were allowed to migrate for 24 h after which the inserts were fixed with methanol, stained with crystal violet, imaged by an inverted microscope and counted by ImageJ [14].

### 2.7. Cytokine-Chemokine Array Analysis

Cytokine array of CCA-CM and LEC/LPS-LEC-CM was performed using the Proteome Profiler Human XL Cytokine Array Kit (R&D Systems, Minneapolis, MN, USA) to determine the cytokine composition of the respective CM. LEC-CM or LPS-LEC-CM was used for overnight membrane incubation, followed by washing, secondary antibody incubation and imaging with Image Quant LAS 4000 system (GE Healthcare, Life Sciences, Chicago, IL, USA) as per manufacturer’s protocol. Densitometric analysis of bands was performed using ImageJ software. Data was normalized using pixel density of reference spots as per the manufacturer’s instructions.

### 2.8. Scratch Wound Assay

CCA cells (HuCC-T1, Mz-ChA-1 and CCLP1) were plated in each well of a 12 well plate. Cells were pretreated with or without CXCR2 inhibitor, SB2250002 (1 μM) for 2 h. A scratch was made with 20 µL pipette tip in each well followed by treatment of CCA cells with either LEC-CM, LPS-LEC-CM, or CXCL5. A picture of the wound was taken at 0 h, 4 h, 6 h, 12 h, 24 h and 48 h. Migration rate was expressed as the average migration rate in µm/h. Wound length was measured by ImageJ [25].

### 2.9. Immunofluorescence (IF) and Immunohistochemistry (IHC)

Tissues isolated for immunofluorescence were frozen in optimal cutting temperature (O.C.T) compound purchased from Tissue-Tek (Sakura-Finetek, Torrance, CA, USA) or were paraffin embedded for IHC. IF was performed with normal and tumor liver and normal and tumor draining liver LN cryosections from control and orthotopic mice with CK19 (cholangiocyte specific marker) and LYVE1/podoplanin (LEC markers). CK19 antibody (#ab52625) was purchased from Abcam (Cambridge, MA, USA), Podoplanin (#NB600-1015) was purchased from Novus Biologicals (Littleton, CO, USA) and LYVE1 (#AF2125) was purchased from R&D systems (Minneapolis, MN, USA). Liver tumor sections from CCA orthotopic and control mice were also stained with CK19 and CXCL5/CXCR2 for checking CXCL5/CXCR2 expression of CCA cells. Sentinel LN sections from CCA orthotopic mice were stained with LYVE1/PDPN and CXCL5. All antibodies were used in 1:100 concentration. At least 3–5 images were taken for all tissue sections. IHC: For IHC with podoplanin and CK19, human CCA arrays were purchased from U.S Biomax (U.S Biomax, Inc., Derwood, MD, USA). Expression of Podoplanin and CK19 was assessed in commercially available paraffin embedded de-identified CCA tumor section arrays containing a total of 70 cases of CCA and 10 normal tissues (U.S Biomax, Inc., Derwood, MD, USA) as described previously [14]. The slides were stained by DAB (3,3′-diaminobenzidine) method with dual-colour DAB staining kit purchased from Vector laboratories (Burlingame, CA, USA).

### 2.10. Tube Formation Assay

LECs (3 × 10^3^) were seeded on top of growth factor reduced Matrigel (BD Biosciences, Franklin Lakes, NJ, USA) in a 96 well plate. The cells were pre-treated with or without SB225002 (1 μM) for 1 h before the addition of CXCL5. Untreated LECs were used as control. Tube formation was then monitored over 6 h, after which the tubes were stained with Calcein AM (2 µM) (Invitrogen, ThermoFisher Scientific, Waltham, MA, USA) and imaged under 4× objective using Olympus fluorescent microscope. The total branching length was measured by the ImageJ software using the Angiogenesis Analyzer plugin [26].

### 2.11. ELISA for CXCL5

LECs were plated in 12 well plates and treated with LPS (100 ng/mL) for 24 h and LEC-CM and LPS-LEC-CM was collected as described above. ELISA was carried out using the Human CXCL5/ENA-78 Quantikine ELISA Kit (R&D Systems, Minneapolis, MN, USA), according to manufacturer’s instructions. Values were represented as pg/mL.

### 2.12. Seahorse Assay

Glycolysis and mitochondrial respiration of CCA cells in response to LEC/LPS-LEC-CM were assessed using the Seahorse XFp Cell Energy Phenotype Test Kit (Agilent Technologies, Santa Clara, CA, USA) in Seahorse XFe96 extracellular flux analyser, according to manufacturer’s protocol. CCA cells (10 × 10^3^) were plated per well in XFe96 plates and incubated with a complete DMEM/RPMI cell culture medium in a 5% CO_2_ atmosphere at 37 °C for 24 h. In this assay, three drugs oligomycin (an ATP synthase inhibitor), rotenone and antimycin A (Rot/AA) (inhibitor of respiratory complex I and II respectively) (Agilent Technologies, Santa Clara, CA, USA) were used to calculate OCR and ECAR. Oligomycin (1.5 μM) and Rot/AA (0.5 μM) were used as per manufacturer’s protocol. The medium was then changed to 5% FBS containing EGM-MV2, LEC-CM, or LPS-LEC-CM for 24 h. After 24 h, the subsequent cell culture medium was replaced with an unbuffered DMEM XF assay medium (pH adjusted to 7.4 using 1N sodium hydroxide) supplemented with 2 mmol/L glutamine and 1 g/L glucose. The cells were kept at 37 °C in a CO_2_-free incubator for 1 h. Basal oxygen consumption rate (OCR) and extracellular acidification rate (ECAR) were consequently determined using the XFe96 plate reader using Wave software version 2.1.6 (Agilent Technologies, Santa Clara, CA, USA), as per manufacturer’s instructions. The OCR at time points before oligomycin and post-Rotenone/Antimycin A injection is associated with basal and maximal respiratory rate, respectively. The ECAR at time points before oligomycin injection and post-oligomycin injection is associated with basal and maximal glycolytic capacity [27,28]. Following an 18 min baseline establishment, OCR, ECAR, or metabolic potential of all groups, was recorded for 48 min based on stressed OCR and stressed ECAR percentage. Normalization of results was done by staining cells with CyQUANT cell proliferation assay kit (ThermoFisher Scientific, Waltham, MA, USA) as described earlier [14,28,29,30]. Data analysis was carried out using the GraphPad Prism software 8 (GraphPad Software, Inc., San Diego, CA, USA).

### 2.13. Lactate and Glucose-Glo™ Assay

For measurement of lactate and glucose levels, lactate and Glucose-Glo™ assay kits were purchased from Promega (Madison, WI, USA). CCA cells were plated in 96 well plate in plain RPMI or DMEM media with no added growth factors overnight. They were then treated with plain EGM (containing 5% FBS), LPS-LEC-CM, plain RPMI/DMEM (containing no FBS), and CXCL5. After 24 h, the cells were treated with lactate and glucose detection reagents and lactate and glucose levels were measured in luminescent mode as per manufacturer’s protocol.

### 2.14. MitoSOX Assay

To measure mitochondrial ROS levels 5 µM MitoSOX reagent (M36008, ThermoFisher Scientific, Waltham, MA, USA) was used to stain CCA cells treated with CXCL5/CXCL5 + SB for 1 h followed by flow cytometry using BD Fortessa ×20 (BD Biosciences, Franklin Lakes, NJ, USA) (Texas A&M University, College of Medicine-Cell Analysis Facility core, Bryan, TX, USA) to detect the number of MitoSOX+ cells. Data analysis was performed using FlowJo software (Ashland, OR, USA).

### 2.15. Statistical Analysis

All data were expressed as mean ± SEM of at least 3 independent experiments unless otherwise stated. Statistical analyses were performed using Prism 8 (GraphPad Software, Inc., San Diego, CA, USA). Differences between groups were analyzed by unpaired Student’s t-test during the analysis of two groups and two-way ANOVA when more than two groups were analyzed, followed by a suitable post hoc test. The level of significance was set at *p* < 0.05.

## 3. Results

### 3.1. Inflamed LECs Enhance Tumor Migration and Activation of Critical EMT Regulators in CCA

**Figure 1:**
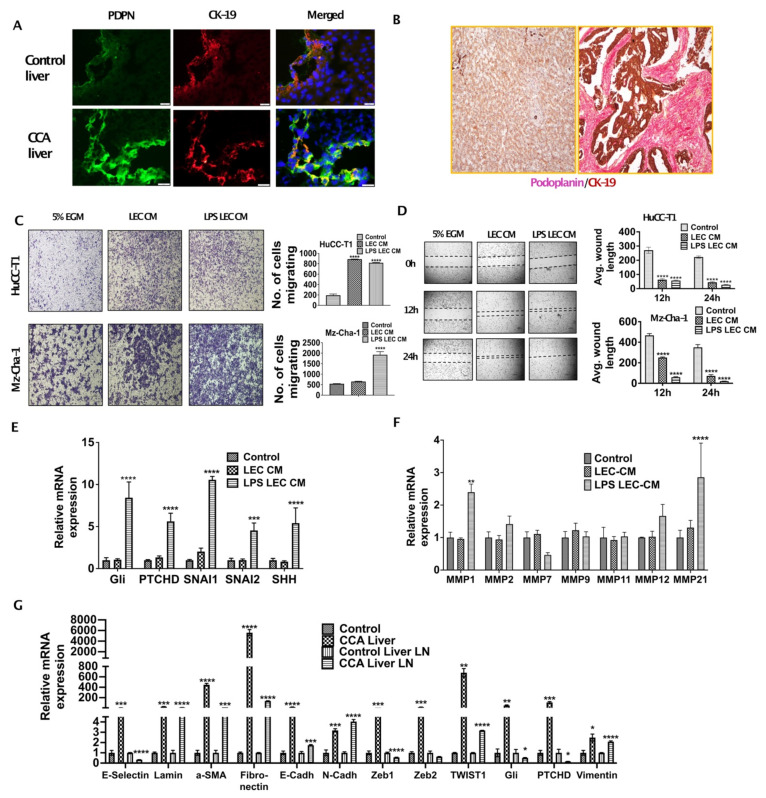
Inflamed LECs enhance tumor migration and activation of critical EMT regulators in CCA. (**A**) Orthotopic syngeneic tumors exhibit increased intratumoral lymphatics. Representative immunofluorescence (IF) images at 40× of Podoplanin expression (green fluorescence) and cytokeratin19 (CK-19, red fluorescence) with DAPI in tumor tissues from orthotopic syngeneic CCA mice (SB1). Scale bar is 20 μm. At least 3–5 images were taken for all tissue sections. (**B**) CCA patients have increased lymphatic invasion in tumor tissues. Representative immunohistochemistry images at 20× magnification of Podoplanin expression (pink) and CK-19 (brown) expression in paraffin embedded sections of CCA patients and in normal liver tissue. (**C**,**D**) Inflamed LEC induces increased migration of CCA cells. (**C**) HuCC-T1 and Mz-Cha-1 cells’ migration towards LEC-CM/inflamed LEC-CM) or 5% EGM for 24 h. Representative images at 10× magnification are shown. Number of cells migrated is plotted. (**D**) HuCCT1 and Mzcha1 cells were grown in 24 well plate and a wound was made on the confluent monolayer. The cells were then treated with LEC/inflamed LEC-CM, or 5% EGM and the average wound length was monitored for 24 h. Representative images at 4× magnification are shown. Average wound length is plotted. Values are represented as mean ± SEM, *n* ≥ 3; **** *p* ≤ 0.0001 represents value significantly different than control (5% EGM). (**E**,**F**) Inflamed LEC induces EMT, matrix remodeling expression in CCA cells. CCA cells were treated with LEC/inflamed LEC-CM or 5% EGM for 24 h. mRNA levels of EMT genes (**E**), *** *p* ≤ 0.001 and **** *p* ≤ 0.0001 value versus control (5% EGM) or MMP genes (**F**) were measured by qPCR. Values are plotted as relative mRNA expression to control and are represented as mean ± SEM, *n* ≥ 3. ** *p* ≤ 0.01 and **** *p* ≤ 0.0001 value versus control (5% EGM). (**G**) EMT genes are induced in SB1 liver and liver LN. mRNA levels of EMT genes in liver and liver LN of control and SB1 mice were quantified by qPCR. Values are plotted as relative mRNA expression compared to control and are represented as mean ± SEM, *n* ≥ 3. * *p* ≤ 0.05, ** *p* ≤ 0.01, *** *p* ≤ 0.001 and **** *p* ≤ 0.0001 represents value versus control (5% EGM).

Increased lymphangiogenesis is associated with poor outcome in CCA. To evaluate the extent of infiltration of CCA cells to LNs and LEC infiltration in CCA TME we stained tumor and liver LN section from orthotopic CCA mice with CK19/Podoplanin and CK19/LYVE1 respectively. We found that a dense network of LECs and lymphatic vessels were present surrounding CCA tumor cells (Figure 1A). A significant increase in lymphatic infiltration, stained by Podoplanin (PDPN) (lymphatic specific marker), was observed in CCA liver as compared to livers from normal controls (Figure 1A). Cholangiocytes were stained by CK19 that also showed a significant increase in expression in the CCA tumor sections. Human CCA arrays were co-stained with Podoplanin and CK19 and high degree of tumor associated lymphangiogenesis was observed (Figure 1B). We hypothesized that chemoattraction and migration might be the first few changes that occur as a consequence of CCA-LEC crosstalk and lead to tumor metastasis to draining LNs.

To determine if LECs enhanced CCA migration, we tested the migration potential of CCA cells by Boyden chamber assay in response to LECs, as well as inflamed LECs, and found that the migration of CCA cells (HuCC-T1 and Mz-ChA-1) was significantly increased in response to CM from both LECs as well as inflamed LECs (*p* ≤ 0.0001) (Figure 1C). We also performed scratch wound assay with CCA cells that were treated with LEC-CM or LPS-LEC-CM or untreated controls and found that LECs, as well as inflamed LECs, caused complete wound closure after 24 h in CCA cells (HuCC-T1, Mz-ChA-1) (*p* ≤ 0.0001) (Figure 1D). Similar results were also observed with another CCA line (CCLP1) (data not shown). In order to determine specific mechanisms that might increase CCA migration, we evaluated the role of EMT regulators, as well as MMPs. Our qPCR data showed that important EMT inducing genes GLI family zinc finger 1 (GLI1), Patched (PTCH1), twist family bHLH transcription factor 1 (TWIST1), SNAI1 and snail family transcriptional repressor 2 (SNAI2) (*p* ≤ 0.001) are induced in CCA cells in response to inflamed LECs (Figure 1E). Treatment of HuCC-T1 with LEC-CM and LPS-LEC-CM for 24 h significantly induced MMP1 and MMP21 in LPS LEC CM treated HuCC-T1, two critical MMP genes (*p* ≤ 0.001) (Figure 1F) thus highlighting a potential role of inflamed LECs in matrix remodeling in CCA that could potentially promote metastasis. These findings were also corroborated in the liver and liver LNs from orthotopic CCA animals that showed a high expression of EMT genes such as *α-Sma*, *Fibronectin*, *Gli*, *Ptchd1*, *Twist1* as well as *Vimentin* indicating activation of the EMT pathways in vivo (Figure 1G). Significant induction was seen specifically for α-*Sma*, *Fibronectin* and *Twist1* in the liver tumor tissues compared to the normal livers from controls.

### 3.2. Inflamed LECs Express High Levels of CXCL5 and Induce the Upregulation of CXCR2 Expression in CCA Cells

**Figure 2:**
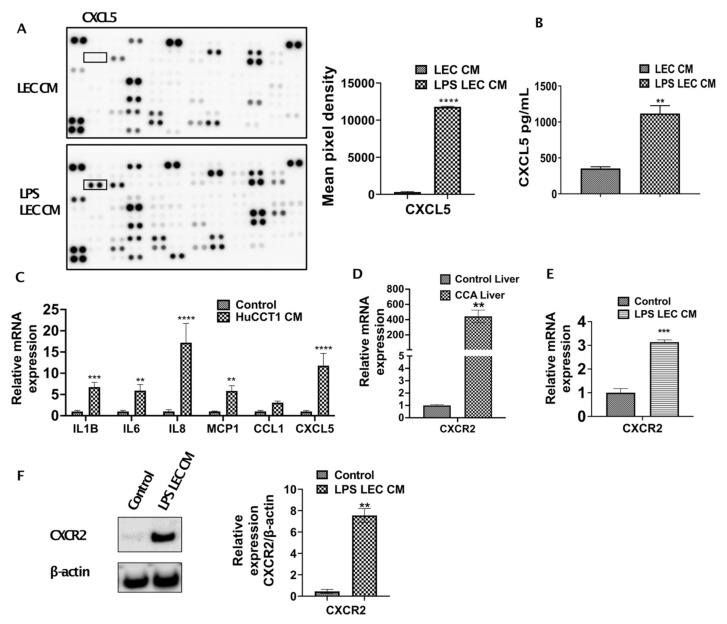
Inflamed LECs express high levels of CXCL5 and induce upregulation of CXCR2 expression in CCA cells. (**A**) Cytokines present in conditioned medium (CM) of LECs primed with LPS for 24 h were analyzed using human cytokine array kit (left panel) and the cytokine, CXCL5 (right panel) was quantified by densitometric analysis of the spots using ImageJ. Values are plotted as mean pixel density and represented as mean ± SEM, *n* ≥ 3; **** *p* ≤ 0.0001 represents value versus LEC CM (**B**) CXCL5 levels in LEC and LPS-LEC-CM were quantified by ELISA. ** *p* ≤ 0.01 value versus control (**C**) LECs were treated with CCA-CM for 24 h and the expression of inflammatory cytokine/chemokines including CXCL5 were measured by qPCR. Values are represented as mean ± SEM, *n* ≥ 3. ** *p* ≤ 0.01, *** *p* ≤ 0.001 and **** *p* ≤ 0.0001 value versus control (5% EGM). Expression of CXCR2 was measured by qPCR in (**D**) livers of control and orthotopic CCA tumor mice, ** *p* ≤ 0.01 value versus control liver and (**E**) CCA cells treated with LPS-LEC-CM. Values are represented as mean ± SEM, *n* ≥ 3. **** *p* ≤ 0.0001 value versus control (5% EGM). (**F**) CCA cells were treated with LPS LEC CM and the protein level expression of CXCR2 was measured by Western blot. Values are represented as mean ± SEM, *n* ≥ 3, ** *p* ≤ 0.01 value versus control.

**Figure 3:**
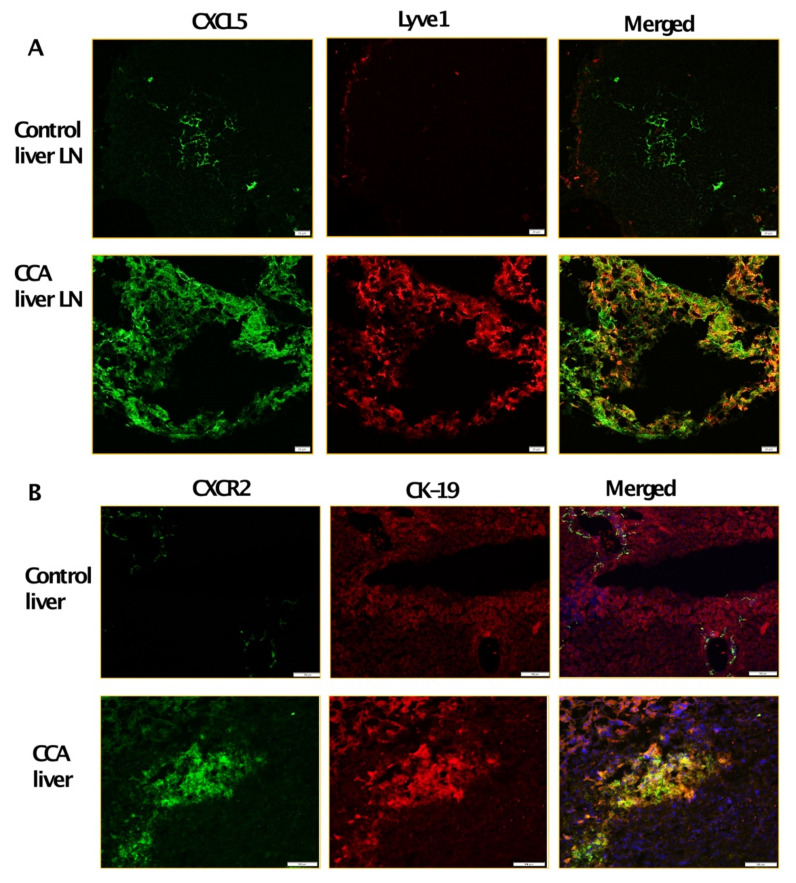
Tumor draining liver LNs and tumor liver express high levels of CXCL5 and CXCR2. Representative immunofluorescent images at 40× magnification of (**A**) CXCL5 expression (green fluorescence) and LYVE1 expression (red fluorescence) in liver LN of control and orthotopic CCA mice. Scale bars are 20 μm. Representative immunofluorescent images at 10× magnification of (**B**) CXCR2 expression green fluorescence) and CK19 expression (red fluorescence) in control liver and tumor from control and orthotopic CCA mice respectively. Scale bars are 100 μm. At least 3–5 images were taken for all tissue sections.

To further determine which cytokines/chemokines expressed by inflamed LECs may cause the observed upregulated CCA migration, we analyzed the LEC-CM and the LPS-LEC-CM by a human cytokine-chemokine profiler array. Among other cytokines and chemokines, CXCL5 was found to be highly expressed in inflamed LEC-CM (*p* ≤ 0.0001) compared to LEC-CM (Figure 2A). To confirm that levels of CXCL5 expression are induced in LPS treated LECs, ELISA was performed. Significantly higher levels of CXCL5 were detected in response to LPS-LEC-CM (Figure 2B) (*p* < 0.001) compared to LEC-CM. Further, corroborating our findings, LECs when treated with CCA-CM also showed increased gene expression of CXCL5 (*p* ≤ 0.0001) in addition to increased expression of critical inflammatory agents implicated in tumor-associated lymphangiogenesis such as IL1B, IL6, IL8 (Figure 2C). Since CXCL5 signals through its cognate receptor CXCR2, we next determined if the expression of CXCR2 is induced by LECs in CCA cells. CXCR2 was found to be significantly elevated in livers from the CCA orthotopic tumor model compared to control animals (*p* < 0.0001) (Figure 2D). Interestingly, we also found that CCA cells when treated with inflamed LPS-LEC-CM showed an increase in both mRNA and protein levels of CXCR2 (*p* ≤ 0.01) (Figure 2E,F). Further, to establish a correlation with these observations in vivo, CXCL5 and CXCR2 expression was also evaluated by immunofluorescence in orthotopic liver LN and liver, respectively. Both liver tumor tissue, as well as tumor draining LN, showed a significantly increased expression of CXCR2 and CXCL5 compared to the normal liver and naïve LNs from the control animals (Figure 3A,B).

### 3.3. CXCL5 Increases Migration and Tumor Promoting Pathways by Inducing Critical EMT Determinants and Focal Adhesion Molecules in CCA and Also Enhance Lymphangiogenesis

**Figure 4:**
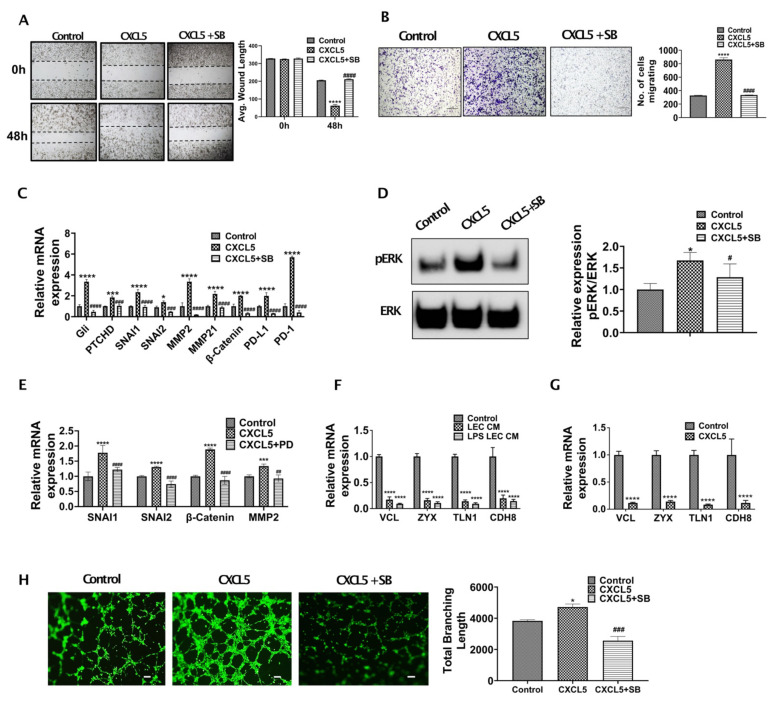
CXCL5 induces critical EMT associated genes in CCA and also enhance lymphangiogenesis. (**A**,**B**) CXCL5 induces increased migration of CCA cells. (**A**) CCA cells were pretreated with or without CXCR2 inhibitor, SB225002 (denoted as SB). A scratch was made on the confluent monolayer. The cells were then treated with CXCL5, and the average wound length was monitored for 48 h. Representative images at 4× magnification are shown. Values are represented as mean ± SEM, *n* ≥ 3. **** represents value significantly different than control group respectively at *p* ≤ 0.0001. #### represents value significantly different than CXCL5 treated group at *p* ≤ 0.0001. (**B**) CCA cells were pretreated with or without SB and were allowed to migrate towards CXCL5 in transwell inserts for 24 h. Representative images at 10× magnification. Scale bars are 100 μm. Number of cells migrated is plotted. **** represents value significantly different than control group respectively at *p* ≤ 0.0001. #### represents value significantly different than CXCL5 treated group at *p* ≤ 0.0001. (**C**) CXCL5 induces expression of EMT, matrix remodeling and immune checkpoint related genes in CCA. CCA cells were pretreated with or without SB and then with or without CXCL5 for 24 h. Expression of EMT, matrix remodeling and immune evasion related genes were measured by real time PCR. Values are represented as mean ± SEM, *n* ≥ 3. * *p* ≤ 0.05, *** *p* ≤ 0.001 **** *p* ≤ 0.0001 represents value significantly different than control group respectively. ### *p* ≤ 0.001 and #### *p* ≤ 0.0001 represents value significantly different than CXCL5 treated group at *p* ≤ 0.0001. (**D**) CXCL5-CXCR2 interaction activates ERK in CCA cells. CCA cells were pretreated with SB and then treated with CXCL5 for 24 h. Untreated groups were used as control. Expression of phospho-ERK and total ERK were measured by western blotting. Densitometric analysis was carried out. Graph represents relative band intensities of pERK/ERK. Values are represented as mean ± SEM, *n* ≥ 3. * represents value significantly different than control and # represents value significantly different than CXCL5 at *p* ≤ 0.05. (**E**) CXCL5 induces expression of EMT and matrix remodeling genes in CCA in an ERK dependent manner. CCA cells were pretreated with or without PD98059 (PD) and then with or without CXCL5 for 24 h. Expression of EMT of matrix remodeling genes were measured by qPCR. Values are represented as mean ± SEM, *n* ≥ 3. *** *p* ≤ 0.001 and **** *p* ≤ 0.0001 value versus control, ## *p* ≤ 0.01 and #### *p* ≤ 0.0001 represents difference from CXCL5 group respectively. (**F**,**G**) Inflamed LECs and CXCL5 alters expression of focal adhesion genes in CCA. CCA cells were treated with (**F**) LEC-CM, LPS-LEC-CM or 5% EGM (control), values are represented as mean ± SEM, *n* ≥ 3. **** *p* ≤ 0.0001 represents value versus control and (**G**) with or without CXCL5 for 24 h and the mRNA levels focal adhesion genes were analyzed by qPCR, Values are represented as mean ± SEM, *n* ≥ 3. **** *p* ≤ 0.0001 represents value versus control. (**H**) CXCL5 induces lymphangiogenesis. LECs were allowed to form tubes on Matrigel with or without CXCL5/CXCL5 + SB and stained with Calcein AM. Representative images in 4× magnification are shown. Total branch length was calculated using ImageJ and plotted. Values are represented as mean ± SEM, *n* ≥ 3. * *p* ≤ 0.05 represents value significantly different than control and ### *p* ≤ 0.001 represents value compared to CXCL5.

To understand if CXCR2-CXCL5 signaling crosstalk alters CCA migration and metastasis, we performed the Boyden chamber assay and scratch wound assay with CCA cells treated with/without CXCL5 and CXCL5 + SB225002 for 24 and 48 h, respectively. A significantly high rate of wound healing (*p* ≤ 0.0001) (Figure 4A) and migration (*p* ≤ 0.0001) (Figure 4B) was observed in CCA cells treated with CXCL5.

To clearly define the mechanistic basis of CXCL5 mediated increased CCA migration and wound healing, we treated CCA cells with CXCL5 and evaluated the expression of EMT and MMP genes. We found that critical EMT determinants such as Gli, PTCHD, SNAI1, SNAI2 were highly expressed (*p* ≤ 0.0001) in CCA cells following treatment with CXCL5 in comparison to CXCL5 + SB treated cells (Figure 4C). We then checked the expression of pERK in CXCL5 treated CCA cells, since ERK has been shown to activate downstream EMT and MMP gene expression thus increasing cancer cell migration in different cancer types. We observed that pERK expression was significantly increased in CXCL5 treated CCA cells (Figure 4D) (*p* < 0.01). We then looked at the expression of key EMT genes known to be regulated by ERK pathway such as SNAI1 and SNAI2 as well as MMP genes in CCA cells treated with CXCL5 and CXCL5 + PD. We found that critical EMT determinants SNAI1 and SNAI2 were highly expressed (*p* ≤ 0.0001) in CCA cells following treatment with CXCL5 in comparison to CXCL5 + PD treated cells (Figure 4E). CXCL5 also induced levels of MMP2 and β-catenin that are associated with matrix remodeling during tumor progression (Figure 4E). These data show that CXCL5-CXCR2 axis operates through ERK pathway to activate EMT and MMP gene expression in CCA cells.

Further, elevated expression levels of focal adhesion genes determine the ability of tumor cells to attach to different tissue sites and form a tumor. As CXCL5 increases CCA migration and activates EMT mechanisms, hence we examined if LEC-CM and CXCL5 had any effect on the focal adhesion genes. CCA cells were treated with CXCL5 for 24 h, and expression of focal adhesion genes was determined. We found significant suppression of several focal adhesion genes such as Vinculin (VCL), Zyxin (ZYX), Talin1 (TLN1), Cadherin 8 (CDH8) (*p* ≤ 0.0001) in CCA cells treated with both inflamed LEC-CM as well as CXCL5, underscoring its role in this axis (Figure 4F,G). These data demonstrate the critical role of CXCL5 in regulating pathways that augment CCA migration.

### 3.4. CXCR2-CXCL5 Signaling Enhances Lymphatic Tube Formation

Metastatic tumor cells promote lymphangiogenesis at the sites of the primary tumor bed [6]. Since inflamed LECs were found to secrete high levels of CXCL5, as well as IL8 (a known lymphangiogenic factor and also binds CXCR2), we wanted to evaluate if the enhanced CXCL5 also induced lymphangiogenesis on inflamed LECs. It was found that LECs treated with CXCL5 showed a significantly higher degree of tube formation in comparison to control (*p* < 0.05) (Figure 4H). These pro-lymphangiogenic effects were significantly abrogated on inhibition of the CXCR2-CXCL5 signaling axis (Figure 4H). This observation also indicates a potential regulatory role of CXCL5 in lymphangiogenesis which becomes important considering the highly lymphangiogenic CCA TME.

### 3.5. Crosstalk with Inflamed LECs Alter Cellular Bioenergetics and Critical Metabolic Gene Expression in CCA Cells

**Figure 5:**
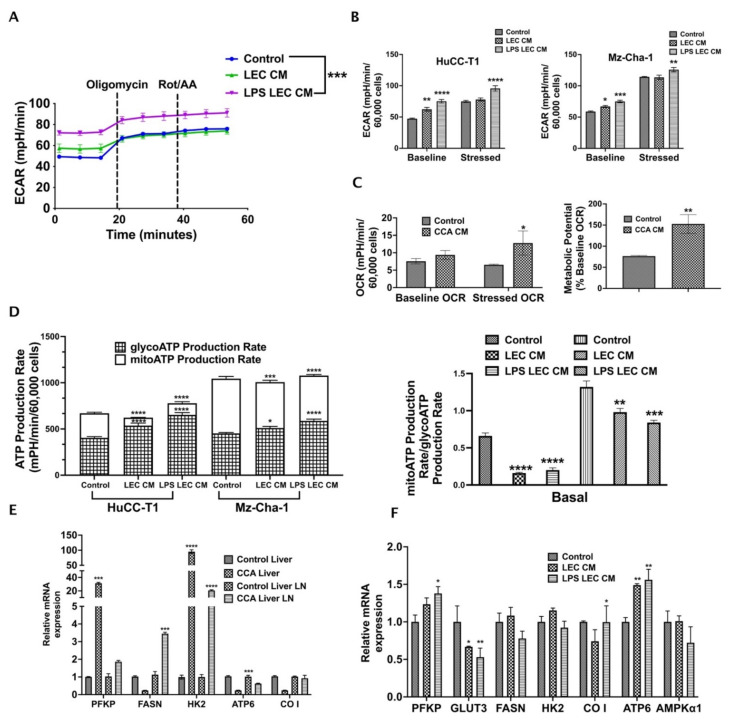
Crosstalk with inflamed LECs alter cellular bioenergetics and critical metabolic gene expression in CCA cells. CCA cells were treated with LEC/LPS-LEC-CM or 5%EGM and Seahorse assay was performed to follow the glycolysis. (**A**) Graph for Extracellular acidification rate (ECAR) of all groups was obtained using Wave software. The time points by which Oligomycin and Rot/AA were injected were depicted in the graphs. The ECAR at time points before Oligomycin injection is associated with glycolysis while that post Oligomycin injection is associated with maximal glycolytic capacity. Values are represented as mean ± SEM, *n* ≥ 3. *** represents value significantly different than control at *p* ≤ 0.001 (**B**) Comparison of baseline vs. stressed ECAR between LEC-CM/LPS-LEC-CM treated CCA cells. * *p* ≤ 0.05, ** *p* ≤ 0.01, *** *p* ≤ 0.001 and **** *p* ≤ 0.0001 represents value significantly different than control. (**C**) Comparison of baseline and stressed OCR in CCA-CM treated LECs. Lower panel, right, shows metabolic potential of CCA-CM LECs. Values are represented as mean ± SEM, *n* ≥ 3. * *p* ≤ 0.05 and ** *p* ≤ 0.01represents value significantly different than control. (**D**) Left panel shows comparison between glycolytic and mitochondrial ATP production rate in LEC-CM/LPS-LEC-CM treated CCA cells. Right panel shows comparison between ATP rate index between LEC-CM/LPS-LEC-CM treated CCA cells. * *p* ≤ 0.05, ** *p* ≤ 0.01, *** *p* ≤ 0.001 and **** *p* ≤ 0.0001 represents value significantly different than control. Expression of metabolic genes were measured by qPCR in (**E**) CCA cells treated with LEC-CM or LPS-LEC-CM. Values are represented as mean ± SEM, *n* ≥ 3. *** *p* ≤ 0.001 and **** *p* ≤ 0.0001 represents value significantly different than control. (**F**) Liver and LNs from CCA orthotopic mice and control animals. Values are represented as mean ± SEM, *n* ≥ 3. * *p* ≤ 0.05 and ** *p* ≤ 0.01 represents value significantly different than control.

Alterations in tumor metabolism is a critical regulator of cancer cell migration and metastasis. To understand the effect of inflamed LECs on CCA metabolism, we performed Seahorse metabolic rate assay with CCA cells treated for 24 h with LEC-CM or LPS-LEC-CM. In this assay, the changes in two critical energy-producing metabolic pathways, i.e., glycolysis and mitochondrial respiration, were quantified as extracellular acidification rate (ECAR) and oxygen consumption rate (OCR) (Figure 5). Interestingly, we found that inflamed LECs significantly alter ECAR or glycolysis of CCA cells (*p* ≤ 0.001) compared to control (Figure 5A). Both baseline (before injection of oligomycin) and stressed (after injection of oligomycin) glycolytic rates were significantly increased in intrahepatic (*p* ≤ 0.0001) and extrahepatic (*p* ≤ 0.001 and *p* ≤ 0.01, respectively) CCA cells treated with inflamed LEC-CM (Figure 5B). Additionally, we also observed that in the presence of CCA-CM the OCR or rate of mitochondrial respiration of LECs was enhanced (Figure 5C) (*p* < 0.01). Consequently, the metabolic potential of LECs, which measures the ability of cells to meet their energy demand via either glycolysis or mitochondrial respiration, was found to be high after exposure to CCA-CM (Figure 5C) (*p* < 0.01). As glycolysis serves as the major source of metabolic fuel for cancer cells, these results show that inflamed LECs alter CCA bioenergetics thereby supplying the energy required to meet the high metabolic demand of CCA cells. In both intra- as well as extra-hepatic CCA, while the total amount of ATP remains almost the same between control and inflamed LEC-CM treated CCA cells, the contribution of glycolysis towards satisfying the total ATP or metabolic energy demand of cancer cells increases significantly (*p* < 0.0001) in basal condition in the inflamed LEC-CM treated CCA group (Figure 5D, left panel). This is interesting because several studies have pointed towards the presence of a distinctly glycolytic phenotype in aggravating cancer metastasis. Further, the ATP rate index (Figure 5D, right panel) shows the ratio of mitoATP (basal rate of ATP production by mitochondrial respiration) to glycoATP (basal rate of ATP production by glycolysis) for each treatment. The lower index of LPS-LEC-CM treated HuCC-T1 and Mz-ChA-1 confirms the altered shift toward glycolysis-based ATP production (Figure 5D, right panel).

Further, the inflamed LECs also significantly increased the expression of critical glucose metabolic genes such as the platelet form of phosphofructokinase 1 (PFKP), cytochrome c oxidase subunit I (CO I), mitochondrially encoded ATP synthase membrane subunit 6 (ATP6) (*p* ≤ 0.05) in CCA cells indicating that ATP synthesis is enhanced (Figure 5E). Confirming these findings in vivo, we observed that in orthotopic CCA mice a number of critical metabolic genes such as Pfkp, fatty acid synthase (Fasn), Atp6 (*p* < 0.001) and hexokinase 2 (Hk2) (*p* < 0.0001) are upregulated in tumor liver and metastatic liver LNs (Figure 5F).

### 3.6. CXCL5 Directly Contributes to the Altered CCA Metabolism and Increases Lactate Production, Glucose Uptake and Induces Mitochondrial Reactive Oxygen Species (mitoROS)

**Figure 6:**
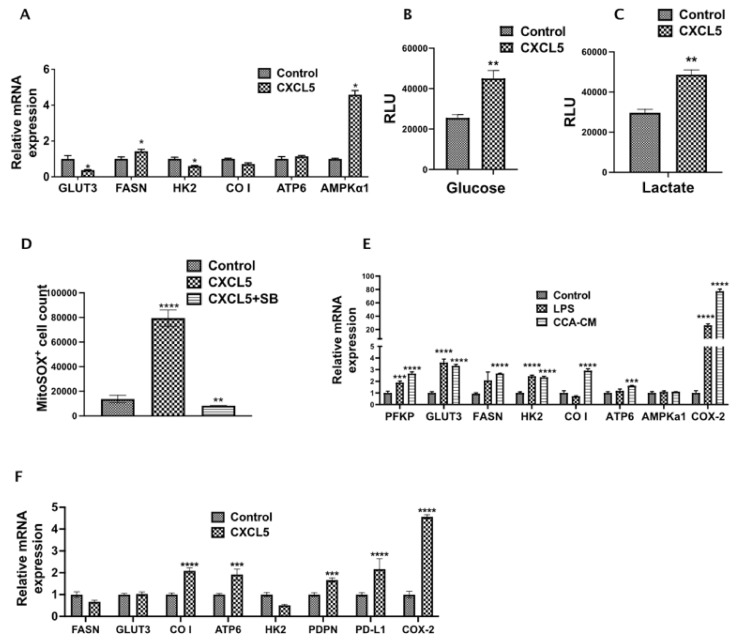
CXCL5 directly contributes to altered CCA metabolism and increases lactate production, glucose uptake and mitochondrial ROS. (**A**). CCA cells were treated with or without CXCL5 for 24 h and the expression of genes involved in glucose and fatty acid metabolism were assessed by qPCR. Values are represented as mean ± SEM, *n* ≥ 3. * *p* ≤ 0.05 represents value significantly different than control. (**B**,**C**) CCA cells were treated with or without CXCL5 for 24 h and the levels of (**B**) glucose and (**C**) lactate were analyzed using Glucose Glo assay and Lactate Glo assay respectively. All values are represented as mean ± SEM, ** *p* ≤ 0.01 represents value versus control. (**D**) CCA cells were pretreated with or without SB and then with or without CXCL5. Levels of reactive oxygen species produced was followed by staining the cells with MitoSOX followed by flow cytometry. ** *p* ≤ 0.01 and **** *p* ≤ 0.0001 represents value significantly different than control (**E**). CCA-LEC crosstalk and inflammation induce metabolic gene expression in LECs. mRNA levels of critical metabolic genes were measured by qPCR. Values represent mean ± SEM, *n* ≥ 3. Values are represented as mean ± SEM, *n* ≥ 3. *** *p* ≤ 0.001 and **** *p* ≤ 0.0001 represents value versus control. (**F**) LECs were treated with CXCL5 for 24 h and the expression of metabolic genes were followed by qPCR as above. All values are represented as mean ± SEM, *n* ≥ 3. *** *p* ≤ 0.001 and **** *p* ≤ 0.0001 represents value versus control.

As alterations in cellular metabolism are tightly linked, we wanted to examine the effects of CXCL5 on CCA metabolism. Interestingly, the expression of crucial metabolic genes such as FASN and AMPKα1 significantly increased in rhCXCL5 treated CCA cells (Figure 6A), indicating some of the observed metabolic changes induced by inflamed LECs are mediated via CXCL5. Further, CCA cells when treated with CXCL5 for 24 h showed a significant increase in the levels of glucose and lactate (*p* < 0.01) (Figure 6B,C). This shows that CXCL5 reprograms and increases the metabolism of CCA cells which may play a role in increased CCA metastasis. Mitochondrial ROS has been shown by previous studies to increase tumor cell survival and proliferation by altering a number of intracellular signaling pathways. We observed that CCA cells treated with CXCL5 for 24 h showed a significant increase in intracellular mitochondrial ROS (*p* < 0.01) (Figure 6D). This observation reveals an important role of CXCL5 in regulating CCA metabolism that may play a part in augmenting CCA metastasis.

### 3.7. CCA-LEC Crosstalk Induce Metabolic Gene Expression in LECs

LEC metabolism is a critical factor in regulating lymphangiogenesis, and cancer cells also express multiple inflammatory mediators that condition cells of TME. Hence, we also evaluated if CCA-CM alter metabolic profile of LECs. We found that the expression level of multiple genes like PFKP, GLUT3, FASN, HK2, CO I, ATP6 was increased (*p* ≤ 0.001) in CCA-CM treated LECs (Figure 6E). FASN has previously been shown to be responsible for high tumor related lymphangiogenesis. To verify if CXCL5 expressed by inflamed LECs had an effect on metabolic and lymphangiogenic potential of LECs directly, we treated LECs with CXCL5 for 24 h and found that there was a significant upregulation of a number of crucial metabolic regulators such as *CO I* (*p* < 0.0001), *ATP6* (*p* < 0.001), lymphatic markers such as *PDPN* (*p* < 0.001), tumor immune evasion marker *PDL1* (*p* < 0.0001), and *COX2* (*p* < 0.0001) (Figure 6F), which has been shown to be involved in tumor lymphangiogenesis in different cancers.

## 4. Discussion

Tumor-lymphatic interactions significantly drive LN metastasis and activation of peri and intratumoral lymphangiogenesis is a key determinant of cancer metastasis [31]. In this study, we report that CXCR2-CXCL5 signaling promotes tumor-LEC interactions in CCA by regulating critical tumor promoting mechanisms, and significantly alters cellular metabolism to further refine an invasive and migratory phenotype of CCA. These mechanisms also act in tandem with upregulation of pro-lymphangiogenic pathways and alteration of LEC bioenergetics that favor a pro-tumorigenic niche. Hence, blocking tumor-lymphatic crosstalk may inhibit the initial tumor spread from the primary tumor bed [6,9]. In this study, we delineate some of the precise mechanisms of tumor-LEC interactions in CCA and illustrate the role of the CXCR2-CXCL5 signaling axis in mediating the crosstalk.

The relationship between liver inflammation and progression of CCA is strongly established by recent studies, and the ensuing liver inflammatory conditions are an important risk factor for CCA [3,32]. In response to an inflamed TME, LECs secrete proinflammatory cytokines/chemokines that further exacerbate the inflammatory milieu, as well as activate several tumor promoting events [33]. A majority of the bacteria-derived endotoxin LPS trafficked through the liver and high levels of LPS are reported in several inflammatory liver diseases such as cholestatic disease, primary sclerosing cholangitis (a premalignant condition and risk factor for CCA) [34]. Cholangiocytes have high Toll-like receptors (TLR4) and LECs have been shown to respond significantly to LPS and produce cytokines and chemokines and cell adhesion molecules [35]. Hence, LPS was used as a primary mediator of inflammation in these studies. Other inflammatory mediators such as TNF-a, IL1β, IL6 or TGF-β are also associated with CCA progression [3]. Interestingly, LPS is shown to significantly induce levels of many of these molecules in the CCA TME. Inflammatory mediators such as IL-6 and TNFα activate a number of tumors promoting pathways such as JAK-STAT, p38 MAPK and Akt resulting in increased cell growth, survival and proliferation [36]. We used an orthotopic mouse model of CCA [20] to determine the degree of lymphatic infiltration and found that in both liver tumors and tumor draining liver LNs, there was significant enhancement of lymphangiogenesis compared to control animals. This data also corroborated with the significant lymphatic infiltration observed in the CCA patient tumor tissue sections. Since migration of cancer cells is a crucial component of metastasis, we investigated changes to CCA migration as the first step in the context of CCA-LEC crosstalk. Enhanced CCA migration was associated with induction in key EMT genes and several crucial EMT genes and inducers of EMT such as *GLI*, *PTCHD1*, *SNAI1*, *SNAI2*, and *SHH* [37], in tumor cells in response to inflamed LECs. Similar activation of the EMT mechanisms was also found in vivo in the liver tumor and tumor draining LN tissues from the CCA orthotopic tumor model. Further, levels of MMPs, *MMP1* and *MMP21* were also induced in response to LECs indicating that LEC-CM also initiated matrix remodeling events. During cancer progression, matrix remodelling and alterations in the expression of MMPs mediates many of the changes in the tumor microenvironment associated with angiogenic or lymphangiogenic processes [38]. Hence, this is a critical finding that LEC-produced factors promote activation of EMT mechanisms and initiate matrix remodelling in CCA that can potentially act as the first step for tumor cell extravasation into lymphatic vessels and subsequent migration to the LN.

Inflamed LECs secrete several cytokines and chemokines that may participate in the crosstalk and elicit the activation of these pathways. We found CXCL5 was significantly elevated in inflamed LECs. This also corelated with its increased mRNA expression in the LECs. In addition, LECs that were exposed to CCA-CM showed a marked induction in CXCL5. Concomitantly, we found that the expression level of CXCR2, the cognate receptor of CXCL5, was highly expressed at both mRNA and protein levels in inflamed LEC-CM treated CCA cells. Activation of several inflammatory genes in LECs such as monocyte chemoattractant protein 1 (MCP1), Interleukin 1 beta (IL1β), interleukin 6 (IL6) and interleukin 8 (IL8) are key players in the process of inflammation, recruitment of immune cells and lymphangiogenesis [39]. This also shows that in the CCA TME, interaction between the tumor cells and the lymphatics promotes an inflammatory signaling cascade that further potentiates tumor progression and sets the stage for increased LN metastasis.

We found that CXCL5 significantly upregulated the migration rate of CCA cells similar to the effects observed by LEC-CM. Activation of this chemokine signaling pathway leads to activation of the pERK pathway, that plays a significant role in regulating cancer progression. It is important to note that ERK activation can play dual roles in cancer in a context-dependent manner by either activating pro-survival or apoptotic mechanisms [40]. Clinical studies also indicate a variable association between ERK activation and tumor progression and could correlate with either an oncogenic or a tumor-suppressing role [41].

Inhibition of CXCR2 diminished the tumor migration to LECs, while suppression of p-ERK also decreased levels of key EMT regulators underscoring the important role of ERK as a downstream regulator of the CXCR2-CXCL5 mediated signaling. Several of these EMT genes were induced in vivo in the liver tumor and sentinel LN isolated from orthotopic CCA mice. In hepatocellular carcinoma (HCC) a number of studies have pointed to the correlation of ERK with multidrug resistance and EMT phenotype [42]. In such studies ERK has been shown to induce transcription of critical EMT-related genes such as SNAI1 [43,44], SNAI2 [44], β-Catenin [45] and MMP2 [46] similar to what we report in these studies. MMP2 is a zinc-dependent endopeptidase that causes the breakdown of a subset of extracellular matrix components during cancer metastasis [47]. Thus, there is a possible mechanistic link between the high degree of lymphangiogenesis observed in CCA and the aggressive CCA metastasis through the Erk-β-Catenin-Snai1-Snai2-Mmp2 pathway. CXCR2 is a key regulator of immune suppression and inflammation in tumor microenvironment and plays crucial role in driving tumor associated macrophage (TAMs) polarization [48]. In multiple cancer types, CXCR2 is also known for recruitment of tumor associated neutrophils (TANs) to the TME [49,50]. Mobilization of neutrophils and monocytes to the tumor tissue bed contributes to tumor growth, support tissue remodeling and contribute to the inflammatory microenvironment [51]. CXCR2 ligands such as CXCL5 also act as potent TAN chemoattractants [52], promoting neutrophil infiltration to sites of inflammation [53,54]. TANs are important in cancer progression and therapeutic resistance by regulating different processes such as angiogenesis, metastasis and immune evasion [55,56,57]. Further, CXCR2 also regulates CD14^+^/CD16^+^ monocyte cells that are precursors of tissue resident macrophages found to be elevated in CCA patients. and have high VEGF that promote tumor angiogenesis and lymphangiogenesis [58]. High levels of CXCL5, which is known for neutrophil recruitment is associated with poor patient prognosis [59]. Hence, further study of LEC-neutrophil crosstalk based on CXCL5-CXCR2 axis and its impact on CCA metastasis may reveal interesting therapeutic targets. It should be noted that IL8, which was significantly induced in LECs in response to CCA-CM, is an important lymphangiogenic regulator and also binds to CXCR2 [9,60]. Therefore, it cannot be ruled out that some of the effects that we observed on blocking CXCR2 could also be due to inhibition of other CXCR2 ligands such as IL8.

Tumor metabolic shifts and EMT progression are critically linked and induce tumor aggressiveness [19]. We observed in our study that inflamed LECs not only affect the EMT determinants through the CXCR2-CXCL5 axis, but also increase the glycolytic rate of CCA cells. Further, CXCL5 modulated the levels of intracellular lactate and increased glucose uptake in the CCA cells. While glucose is a critical metabolic substrate in tumor cells, lactate is a by-product of the anaerobic phase of glycolysis. Lactate has been found to drive a number of key pro-tumorigenic processes such as angiogenesis, immune evasion, and cell migration. In cancer tissues, a 10–30 mM concentration of lactate has been detected [61]. Lactate is used continuously as a fuel by different cells and plays an important role as an onco-metabolite and activates oncogenes and transcription factors [62,63]. Lactate secreted from tumor cells stimulate VEGFR expression, thus promoting angiogenesis [64,65,66]. Correlation studies have shown that lactate levels are related to a degree of cancer metastasis in a concentration-dependent manner [67]. Lactate has also been shown to activate transforming growth factor-β2 (TGFβ2), which then increases glioma cell migration [68]. In terms of immune evasion, lactate inhibits monocyte migration to the TME and suppresses releases of cytokines such as interleukin 6 (IL6) and tumor necrosis factor (TNF) [67]. Lactate also regulates its immunosuppressive functions in T cells by reducing cytokine production (up to 95%) and cytokine activity (up to 50%) [69]. Thus, the increase in intracellular lactate observed in CCA cells post CXCL5 treatment can have important implications in terms of CCA angiogenesis and lymphangiogenesis, as well as immune evasion.

The increased levels of lactate and glucose brought about by CXCL5 suggests a high rate of glycolysis in CCA that supports CCA migration by providing the requisite metabolic fuel. This effect on cell metabolism arising from intercellular crosstalk between CCA and LECs also showed significant changes in the mRNA expression level of crucial metabolic genes such as PFKP, GLUT3, FASN, HK2 and CO I in LECs treated with CCA-CM. Thus, EMT-mediated changes in CCA metastasis that are regulated by changes in tumor metabolism caused by inflamed LECs is a key finding of this study. One of the metabolic genes we found induced in both orthotopic CCA liver, as well as inflamed LEC-CM treated CCA cells, was platelet isoform of the rate-limiting allosteric enzyme of glycolysis phosphofructokinase-1 (PFKP). PFKP shows abnormal expression in many cancers and augments lactic acid production due to a high rate of glycolysis [70,71]. We also observed CXCL5 treatment induced high levels of intracellular lactate in the CCA cells (Figure 6C). Another metabolic regulator, FASN promotes proliferation, migration and survival of HLECs and is a key mediator of tumor lymphangiogenesis [72]. *Fasn* showed significant induction in orthotopic CCA sentinel LN or liver LN (Figure 5F), as well as in CCA-CM treated LECs (Figure 6E). We observed that although *Fasn* expression in orthotopic CCA liver remained unchanged compared to control, the metastatic sentinel liver LNs showed a high FASN expression thus supporting the role of FASN in promoting lymphangiogenesis and CCA LN metastasis. This is significant as FASN is known to upregulate LN metastasis through the differential regulation of VEGF-C/D expression in cancer cells and LECs [72]. It should be noted that while treatment of LECs with CCA CM upregulates *FASN* expression (Figure 6E), CXCL5 treated LECs show a decreased trend of FASN expression. LECs secrete several cytokines and chemokines in response to inflammation, as shown in Supplementary Materials, Figure S1.

Similarly, tumor cells also secrete different molecules that significantly impact the TME and tumor metabolism and could account for the enhanced expression of FASN expression in LECs. It is possible that CXCL5 by itself is not sufficient to significantly induce FASN expression in the LECs, although it is significantly induced in response to other contributing factors. We noted overexpression of another crucial rate-limiting glycolytic enzyme hexokinase 2 (HK2) that has been correlated with poor patient prognosis and aberrant expression patterns in several cancers as hepatocellular carcinoma and others [73]. FDA-approved orphan drug 3-bromopyruvate which blocks glycolysis in cancer cells by inhibiting HK2 enzymatic activity has shown promising results in HCC patients [74]. Upregulation of *Hk2* expression was noted not only in orthotopic CCA liver and liver LN (Figure 5F), but also in CCA-CM treated LECs, indicating a role of HK2 in CCA associated lymphangiogenesis as well. Another metabolic gene that showed significant induction in CCA-CM and CXCL5 treated LECs was cyclooxygenase 2 (COX2) (Figure 6E,F). COX2 derived prostaglandins stimulate lymphangiogenesis in inflammatory background such as secondary lymphedema [75]. Tumor growth, tumor associated lymphangiogenesis and LN metastasis in breast cancer [76] and lung cancer [77] are regulated by COX2-VEGF-C pathway. Thus, COX2 can emerge to be a promising target in mitigating early aggressive LN metastasis commonly observed in CCA. Additionally, an upregulation of *Atp6* was also found in orthotopic CCA liver (Figure 5F), inflamed LEC-CM treated CCA (Figure 5E) and inflamed LEC-CM and CXCL5 treated LECs (Figure 6E,F). ATP6 is a mitochondrial gene encoding F_0_ subunit 6 of ATP synthase enzyme involved in mitochondrial respiration [78]. Its role in cancer metastasis is still understudied, however, mitochondrial DNA mutations have been linked to tumor growth and metastasis. ATP6 gene is more prone to be mutated in breast cancer patients among mitochondrial genes [79], leading to metabolic alterations.

The effect of LECs on the overall alteration in CCA metabolism, as observed in this study, sheds some interesting insights by suggesting that LECs alter key tumorigenic mechanisms that predispose metastasis and could be important targets for therapeutic intervention. The CCA cells undergo further metabolic reprogramming in the presence of LECs and LEC mediated factors that fuels tumor growth. In the LECs, significant alterations in cellular bioenergetics predispose a pro-lymphangiogenic signaling that further promotes tumor migration into lymphatics and subsequent LN metastasis. Such studies point toward the need to study new lymphatic targets for the development of anti-lymphangiogenic therapy in CCA. Specific molecular targets such as CXCR2 and specific metabolic regulators identified in this study can thus be targeted, and the role of LECs in CCA can emerge to be a significant factor contributing to the aggressive metastasis in this disease.

## 5. Conclusions

**Figure 7:**
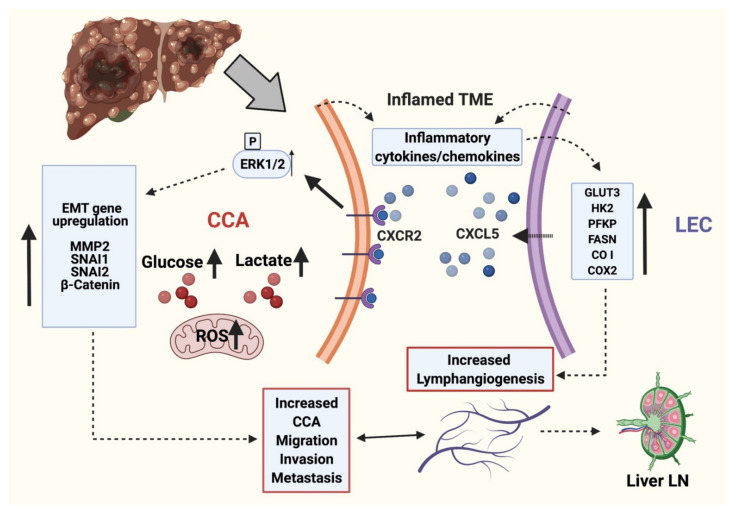
Working model of molecular interplay between LEC and CCA during CCA progression. LECs and CCA cells in inflamed TME actively produce several cytokines and chemokines. CXCL5 produced by inflamed LECs interacts with CXCR2 in CCA cells and potentiates EMT, matrix remodeling and alters cellular metabolism. The proinflammatory cytokines and chemokines in the TME in turn induces metabolic alterations in LECs resulting in ATP generation and activation of lymphangiogenesis. This signaling axis potentiates CCA LN metastasis. Thus CXCL5-CXCR2 crosstalk between LECs and CCA cells in inflamed environment results in aiding the lymphatic metastasis of CCA cells. (This schematic was created with BioRender.com accessed on 25 October 2021).

In summary, our data suggests that interaction of CCA cells and LECs activates the CXCR2-CXCL5 pathway that promotes tumor-LEC crosstalk, enhances tumor migration, and activates significant EMT modulators, thereby setting the stage for local invasion (Figure 7). These alterations are associated with LEC and CXCL5 induced alterations in CCA metabolism and upregulation of glycolytic pathways, production of lactate and mitoROS, along with significant observed changes in mitochondrial and metabolic gene expression. Suppression of the CXCR2-CXCL5 axis also inhibits pro-lymphangiogenic pathways and could thus be an effective therapeutic target. The identified mechanisms and pathways could lead to novel intervention strategies targeting tumor-lymphatic crosstalk, thereby opening new therapeutic avenues for tumors migrating through lymphatics.

## Figures and Tables

**Table 1:** List of Primers used in this study.

**Human Primers**	**Forward Sequence**	**Reverse Sequence**
*Gli*	AGGGCTGCAGTAAAGCCTTCA	CTTGACATCTTTTCGCAGCG
*PTCHD*	TGAGACTGACCACGGCCTG	ACCCTCAGTTGGAGCTGCTTG
*SNAI1*	CAGACCCACTCAGATGTCAA	CATAGTTAGTCACACCTCGT
*SNAI2*	ACATTAGAACTCACACGGGGA	GTGTGCTACACAGCAGCCAGA
*SHH*	CCCAATTACAACCCCGACATC	TCACCCGCAGTTTCACTCCT
*MMP1*	ACAGCCCAGTACTTATTCCCTTTG	GGGCTTGAAGCTGCTTACGA
*MMP2*	GCTGGCTGCCTTAGAACCTTTC	GAACCATCACTATGTGGGCTGAGA
*MMP7*	GGGACATTCCTCTGATCCTAATGC	GAATTACTTCTCTTTCCATATCGTTTCTGAATGC
*MMP9*	GCACGACGTCTTCCAGTACC	GCACTGCAGGATGTCATAGGT
*MMP11*	CAACATACCTCAATCCTGTCCC	CAATGGCTTTGGAGGATAGC
*MMP12*	TTGAATATGACTTCCTACTCCAACG	GTGGTACACTGAGGACATAGCAAAT
*MMP21*	AACAATAGGACACGCTATGG	CATCTCTTTTCCATGTCCAG
*IL1* *β*	AATCTGTACCTGTCCTGCGTGTT	TGGGTAATTTTTGGGATCTACACTCT
*IL6*	GTAGCCGCCCCACACAGA	CATGTCTCCTTTCTCAGGGCTG
*IL8*	GTGCAGTTTTGCCAAGGAGT	TTATGAATTCTCAGCCCTCTTCAAAAACTTCTC
*MCP1*	ACTCTCGCCTCCAGCATGAA	TTGATTGCATCTGGCTGAGC
*CCL1*	GGAAGATGTGGACAGCAAGAGC	TGTATGGCTGTTAGTTTCGG
*CXCL5*	TGGACGGTGGAAACAAGG	TGGACGGTGGAAACAAGG
*CXCR2*	CACCGATGTCTACCTGCTGA	CACAGGGTTGAGCCAAAAGT
*β-Catenin*	AAAATGGCAGTGCGTTTAG	TTTGAAGGCAGTCTGTCGTA
*PD-L1*	ACAGCTGAATTGGTCATCCCA	CACATCCATCATTCTCCCTTTTC
*PD1*	CGTGGCCTATCCACTCCTCA	ATCCCTTGTCCCAGCCACTC
*VCL*	TCAGATGAGGTGACTCGGTTGG	GGGTGCTTATGGTTGGGATTCG
*ZYX*	GCAGAATGTGGCTGTCAACGAAC	TGAAGCAGGCGATGTGGAACAG
*TLN1*	TTGGAGATGCCAGCAAGCGACT	CCAGTTCTGTGGCTGCCTGATT
*CDH8*	CTACTGAAATTAGGAACCACAGTCAGAT	CTAACAGTTTGAATGACTTGGCCG
*PFKP*	CGGAAGTTCCTGGAGCACCTCTC	AAGTACACCTTGGCCCCCACGTA
*GLUT3*	ACTTTGACGGACAAGGGAAATG	ACCAGTGACAGCCAACAGG
*FASN*	CGCGTGGCCGGCTACTCCTAC	CGGCTGCCACACGCTCCTCT
*HK2*	GAGCCACCACTCACCCTACT	CCAGGCATTCGGCAATGTG
*CO I*	CTCTTGCGGTACTCATTGAAG	GAGCTGCTGTTCGGTGTC
*ATP6*	GAAGCGCCACCCTAGCAATA	GCTTGGATTAAGGCGACAGC
*AMPK* *α1*	TGCGTGTACGAAGGAAGAATCC	TGTGACTTCCAGGTCTTGGAGTT
*COX2*	GAATGGGGTGATGAGCAGTT	CAGAAGGGCAGGATACAGC
*RPL19*	GGGCATAGGTAAGCGGAAGG	TCAGGTACAGGCTGTGATACA
*Ubiquitin*	AGTCCCTTCTCGGCGATTCT	GCATTGTCAAGTGACGATCACAGC
**Mouse Primers**	**Forward Sequence**	**Reverse Sequence**
*E-Selectin*	GGACACCACAAATCCCAGTCTG	TCGCAGGAGAACTCACAACTGG
*Lamin*	GAGCAAAGTGCGTGAGGAGTTC	CCTTGGAGTTGAGAAGAGCCTC
*α-SMA*	TGCTGACAGAGGCACCACTGAA	CAGTTGTACGTCCAGAGGCATAG
*Fibronectin*	CCCTATCTCTGATACCGTTGTCC	TGCCGCAACTACTGTGATTCGG
*E-Cadh*	GGTCATCAGTGTGCTCACCTCT	GCTGTTGTGCTCAAGCCTTCAC
*N-Cadh*	CCTCCAGAGTTTACTGCCATGAC	CCACCACTGATTCTGTATGCCG
*Zeb1*	ATTCAGCTACTGTGAGCCCTGC	CATTCTGGTCCTCCACAGTGGA
*Zeb2*	GCAGTGAGCATCGAAGAGTACC	GGCAAAAGCATCTGGAGTTCCAG
*Twist1*	AGCGGGTCATGGCTAACG	AGCGGGTCATGGCTAACG
*Gli*	CCCATAGGGTCTCGGGGTCTCAAAC	GGAGGACCTGCGGCTGACTGTGTAA
*Ptchd*	TTGAATATGACTTCCTACTCCAACG	GTGGTACACTGAGGACATAGCAAAT
*Cxcr2*	TGTCTGCTCCCTTCCATCTT	CCATTTCCTCTCCTCCAGCT
*Pfkp*	GGAAGCCAAATGGGACTGT	CGCACTACCGATGATGGTC
*Fasn*	CTGCGTGGCTATGATTATGG	AGGTTGCTGTCGTCTGTAGT
*Hk2*	TGGGTTTCACCTTCTCGTTC	TTCACCAGGATGAGTCTGAC
*Atp6*	TCCCAATCGTTGTAGCCATCA	AGACGGTTGTTGATTAGGCGT
*Co I*	ATCACTACCAGTGCTAGCCG	CCTCCAGCGGGATCAAAGAA
*Rpl19*	ATGAGTATGCTCAGGCTACAGA	GCATTGGCCGATTTCATTGGTC
*Ubiquitin*	GCCCAGTGTTACCACCAAGAAG	GCTCTTTTTAGATACTGTGGTGAGGAA

## Data Availability

Not applicable.

## Supplementary Materials

The following are available online at https://www.mdpi.com/article/10.3390/cells10113093/s1, Figure S1: Differential expression of chemokines and cytokines in LECs in response to LPS mediated inflammation.
