# Supplementary material for: Tumor Lymphatic Interactions Induce CXCR2-CXCL5 Axis and Alter Cellular Metabolism and Lymphangiogenic Pathways to Promote Cholangiocarcinoma
Source: Cells. 2021 Nov 9;10(11):3093. doi: 10.3390/cells10113093 (PMC8623887; doi:10.3390/cells10113093)
Supplement: Supplementary file 1 [file cells-10-03093-s001.zip › cells-1339031-supplementary.pdf]

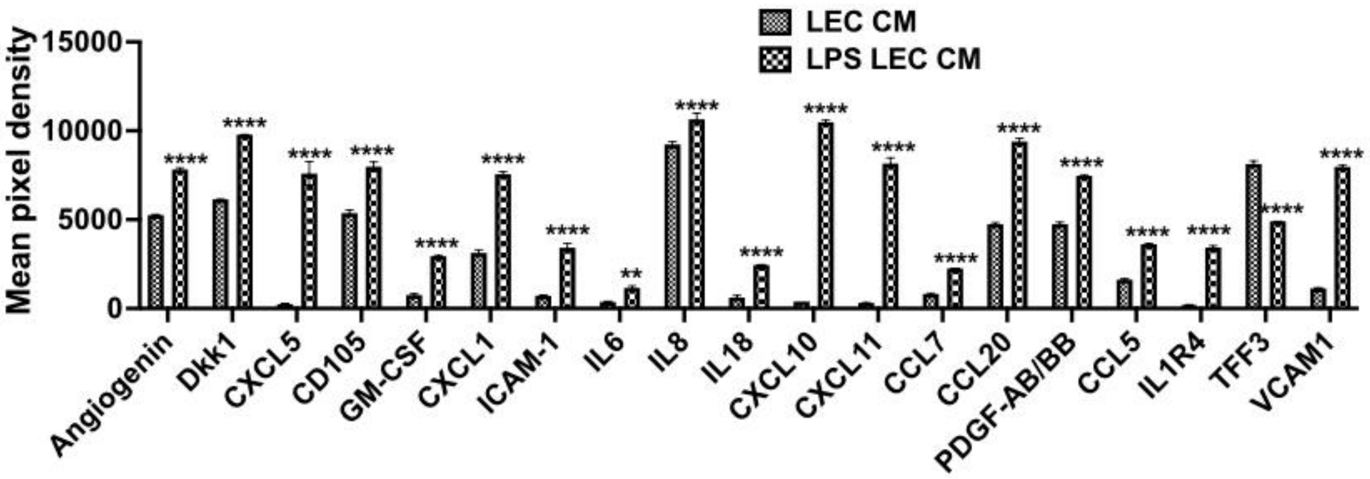


**Supplementary Figure S1.** Differential expression of chemokines and cytokines in LECs in response to LPS mediated inflammation. Cytokines, chemokines and molecules involved in angiogenesis and lymphangiogenesis present in conditioned medium (CM) of LECs primed with or without LPS for 24 h were analyzed using the human cytokine array (R&D Systems) and the cytokines were quantified by densitometric analysis of the spots using ImageJ. Values are plotted as mean pixel density. ** $p \leq 0.01$ and **** $p \leq 0.0001$ represents value significantly different compared to LEC-CM.
